# Clinical characteristics and treatment of hepatic portal venous gas: case series and literature review

**DOI:** 10.3389/fmed.2025.1540418

**Published:** 2025-05-09

**Authors:** Hao-Ran Wei, Yu-Qiang Shan, Fan-He Dong, Lin-Po Zhou, Ye-Bin Yang, Jing Shi, Cheng-Hao Ji, Wen-Cheng Kong

**Affiliations:** ^1^The Fourth School of Clinical Medicine, Zhejiang Chinese Medical University, Zhejiang, China; ^2^Affiliated Hangzhou First People’s Hospital, School of Medicine, Westlake University, Zhejiang, China

**Keywords:** hepatic portal venous gas, clinical characteristics, treatment, prognosis, literature review

## Abstract

**Background:**

Hepatic portal venous gas (HPVG) is often regarded as a critical warning sign and has poor patient prognosis if appropriate treatment measures are not promptly administered. There is significant heterogeneity in clinical manifestations, diagnostic approaches, and outcomes among patients with HPVG; hence, this study aimed to analyze the clinical characteristics of patients with HPVG and explore more effective treatment methods to provide valuable references for future clinical treatment strategies.

**Methods:**

A total of 21 patients diagnosed with HPVG using computed tomography at the First People’s Hospital of Hangzhou between January 2014 and October 2024 were retrospectively analyzed. A comprehensive analyses of the sex, age, laboratory test results, reasons for admission, comorbidities, treatment methods, and outcomes of patients were done.

**Results:**

The mean age of the 21 patients (13 men and 8 women) was 61.7 years. Patients presented with decreased red blood cell and hemoglobin counts, and increased white blood cell, neutrophil, C-reactive protein, and D-dimer levels. The main etiologies of HPVG were peritonitis (52.4%), post-abdominal surgery (47.6%), intestinal necrosis (33.3%), and gastrointestinal bleeding (28.6%), while the common comorbidities were peritonitis (52.4%), hypertension (52.4%), and coronary heart disease (23.8%). The overall mortality rate of patients with HPVG was 28.6%, and most of the deceased patients had bowel necrosis. Platelet count [odds ratio (OR): 0.979; 95% confidence interval (CI): 0.962–0.997; *p* = 0.024] and neutrophil levels, (OR: 1.161; 95% CI: 1.019–1.323; *p* = 0.025), and the presence of hypertension (OR: 15.750; 95% CI: 1.424–174.246; *p* = 0.025) and peritonitis (OR: 15.750; 95% CI: 1.424–174.246; *p* = 0.025) were significantly associated with the likelihood of requiring surgical intervention. Most patients had a good prognosis after surgical treatment.

**Conclusion:**

This study systematically described the clinical characteristics, etiologies, comorbidities, and prognosis of patients with HPVG and identified predictors indicating the need for surgical intervention.

## Introduction

Hepatic portal venous gas (HPVG) is a radiological sign associated with various diseases and is characterized by the radiological appearance of abnormal gas accumulation in the portal vein and its intrahepatic branches (1). This phenomenon was first reported by Wolfe and Evans (2), and primarily occurs when gas from the intestinal lumen or gas produced by certain bacteria enters the portal venous circulation, which makes it a rare radiological finding. The presence of HPVG is considered a marker of serious underlying diseases (3). In a retrospective study of 60 patients with HPVG in 1978, the mortality rate was 75%, and was more prevalent among patients with severe bowel injuries, including severe intestinal ischemia and enteritis (3, 4); hence, it is crucial to pay close clinical attention to this condition. With the widespread adoption and advancement of radiological techniques, the detection rate of HPVG has increased in patients without urgent conditions (5, 6).

Traditionally, resection of the affected area has been believed to be the only effective treatment for HPVG, especially in cases associated with severe underlying conditions, such as bowel ischemia or necrosis; however, advanced imaging techniques, including computed tomography (CT), have shown that some patients can recover from HPVG with non-surgical conservative treatment (7–10). In recovered patients, HPVG is often not associated with bowel necrosis, indicating that not all patients require surgery (11, 12). Furthermore, high-risk surgeries performed in emergency situations are unsuitable for all patients with acute intestinal injuries. Patients with certain congenital conditions, such as Hirschsprung disease or malrotation, poor overall health, severe weakness, or extreme fatigue may not be eligible for surgical intervention (13). With the rapid development of diagnostic tools and treatment methods, the clinical features and management strategies for HPVG have undergone significant changes. Advanced imaging techniques now allow for more accurate diagnosis and risk stratification, and non-surgical, conservative treatments have become viable options for many patients, reducing the need for high-risk surgeries. Previous studies of HPVG have typically been presented as case reports, which highlight a wide range of clinical symptoms, treatment approaches, and outcomes; however, because of substantial heterogeneity among patients, they lack representativeness and generalizability (14–16). The specific criteria and predictors that can help determine which patients are most likely to benefit from surgical management and the optimal treatment strategies for these patients require further elaboration. This study systematically reviewed the clinical symptoms, etiology, treatment methods, and outcomes of patients with HPVG to provide a more personalized and accurate basis for the treatment of these patients.

## Methods

### Patients

This retrospective analysis examined 21 adult patients diagnosed with HPVG at Hangzhou First People’s Hospital between January 2014 and October 2024. Using the hospital search engine to retrieve CT reports containing cases of intrahepatic and/or portal venous gas, we found clear gas accumulation within the hepatic portal venous system. This gas accumulation typically presents in a branching pattern and extends to the subcapsular region of the liver. The CT images showed that the gas formed distinct linear or branching low-density shadows in the main trunk and branches of the portal vein. These low-density areas represent the abnormal gas accumulation within the hepatic portal venous system, which is characteristic of HPVG. The gas can diffuse into small vessels within the liver parenchyma and sometimes appears in the sub-capsular region of the liver, presenting as well-defined low-density areas for gas accumulation. Furthermore, this gas can diffuse into small vessels within the liver parenchyma, and can sometimes appear in the subcapsular region of the liver, forming well-defined areas for gas accumulation. These imaging findings not only indicate the presence of HPVG but also provide detailed insights into the underlying pathophysiological processes of the disease, which can cause bowel wall injuries, such as ischemia or necrosis, bacterial infections, such as *Clostridium perfringens* or *Escherichia coli*, and other mechanisms that allow gas to enter the portal venous system, such as increased intraluminal pressure or mucosal disruption. While relying on retrospective CT reports, there is an inherent risk of radiological error in reporting. The radiologists’ interpretations at the time of diagnosis might be influenced by various factors, and some cases of HPVG could potentially be missed. However, we attempted to mitigate this risk by cross-referencing the imaging reports with comprehensive clinical and laboratory data. The Ethics Review Committee of the Fourth Clinical Medical College of Zhejiang Chinese Medical University approved this study and waived the requirement for informed consent due to the retrospective nature of the study.

### Data collection

By reviewing the medical records of patients, we created a case report form to systematically collect the following demographic and clinical information for each patient, which included the following: (1) demographic information, including sex and age; (2) clinical features, including clinical symptoms, etiology, and comorbidities; (3) laboratory test results, including complete blood count, liver function, kidney function, coagulation profile, and abdominal CT findings; and (4) treatment methods and patient outcomes. To reduce the potential confounding effect of end-stage malignancies, we also performed a separate analysis excluding patients with advanced cancer. Advanced cancer can introduce various systemic changes that may not be directly related to HPVG but could influence the variables under study, such as blood counts and inflammatory markers. By excluding these cases, we aimed to provide a more focused analysis of the relationships between HPVG-related factors, treatment modalities, and patient outcomes. In addition to the demographic and clinical information, for the diagnosis of intestinal necrosis, we considered multiple factors. CT reports of patients typically showed gas in the gastrointestinal wall, intestinal wall edema, abdominal wall edema, thickening and haziness of the peritoneum, mesentery, and omentum, along with fluid in the abdominal and pelvic cavities. CT-reporting physicians indicated a high probability of intestinal necrosis based on these imaging features. Clinically, patients presented with manifestations such as melena and signs of extensive peritonitis. In some cases, abdominal paracentesis was performed, and the aspiration of bloody fluid from the abdominal cavity further supported the diagnosis of intestinal necrosis. For patients with non-end-stage malignant tumors, the decision for surgical or conservative management was based on the interaction between the tumor-related factors (stage, location, and potential impact on the gastrointestinal tract) and the severity of HPVG-associated symptoms. If the tumor was likely to be the root cause of HPVG and was causing critical gastrointestinal complications, surgery was more likely to be performed. Otherwise, conservative treatment was initially explored. The timing of melena onset was a key determinant in the treatment decision. Acute melena with associated hemodynamic instability or rapid hematological deterioration indicated a higher likelihood of surgical intervention to address the bleeding source. Chronic or intermittent melena, in the absence of severe symptoms, led to a preference for initial conservative evaluation and management, such as endoscopic examination and medical treatment. Abdominal pain was a key symptom evaluated in the treatment decision-making. The severity of pain was assessed using a numerical rating scale, and its response to conservative treatment was closely monitored. In cases where the pain score remained high despite 24–48 h of conservative management, and was associated with other signs such as peritonitis or suspected intestinal ischemia, surgical intervention was strongly considered. Relief of abdominal pain with conservative treatment was often an indication that the patient might respond well to non-surgical management.

### Statistical analysis

Categorical variables were presented as numbers and percentages, whereas quantitative data were expressed as mean (range) or median/interquartile range, depending on the data distribution. Differences between the surgical and nonsurgical groups were compared using the chi-square test, Fisher’s exact test, independent *t*-tests, or rank-sum test, depending on the data type and distribution. Logistic regression was applied to identify the predictors of surgery for HPVG, and the effect estimate was reported as odds ratios (OR) with 95% confidence intervals (CI). A two-sided *p* < 0.05 was considered statistically significant. Statistical analyses were performed using SPSS (version 26.0; IBM, Armonk, NY, USA).

## Results

### Baseline characteristics

Of the 21 patients, there were 13 males and 8 females, with a mean age of 61.7 years. The patient demographics are shown in Table 1. We found that the levels of red blood cells (RBC) and hemoglobin (HB) decreased, whereas the levels of white blood cells (WBC), neutrophils, C-reactive protein (CRP), and D-dimer (DD-I) increased.

**Table 1:** The characteristics of 21 patients with HPVG.

Characteristic	Value	Normal value
Total	21	-
Sex (Male/Female, %)	13 (61.9)/8 (38.1)	-
Age (years)	61.7 (16–92)	-
Male/Female	58.9 (18–85)/66.1 (16–92)	-
RBC (Male/Female, 10^12^/L)	4.0 (2.4–6.0)/3.3 (2.4–4.0)	4.3–5.8/3.8–5.1
WBC (10^9^/L)	9.6 (2.2–21.4)	3.5–9.5
PLT (10^9^/L)	183.3 (47–305)	125–350
HB (Male/Female, g/L)	109.4 (69–160)/98.1 (71–120)	130–175/115–150
N (Neutrophil, %)	81.1/21.3	40–75
CRP (mg/L)	61.7/99	0–8
PCT (ng/L)	0.22/0.64	0–0.5
DD-I (D-Dimer, mg/L)	4270/5458	0–500
ALT (U/L)	22/72	9–50
AST (U/L)	37/84	15–40

HPVG, hepatic portal venous gas; RBC, red blood cell; WBC, white blood cell; PLT, platelet; HB, hemoglobin; N, neutrophil; CRP, C-reactive protein; PCT, procalcitonin; DD-I, D-Dimer; ALT, alanine aminotransferase; AST, aspartate aminotransferase.

### Etiologies and comorbidities

The causes and comorbidities of HPVG are shown in Table 2. The most common cause was peritonitis (52.4%), followed by post-abdominal surgery (47.6%), intestinal necrosis (33.3%), gastrointestinal bleeding (28.6%), gastrointestinal tumors (14.3%), intestinal obstruction (14.3%), and abdominal infections (9.5%). Here, peritonitis includes both primary and secondary cases. We categorized factors such as intestinal necrosis, gastrointestinal bleeding, gastrointestinal tumors, and intestinal obstruction separately despite their potential to cause secondary peritonitis. This is because these conditions can independently contribute to the development of HPVG through their own unique pathophysiological pathways. For example, intestinal necrosis can directly lead to gas production within the necrotic area that may enter the portal venous system, and gastrointestinal bleeding can cause local tissue injury and inflammation promoting gas entry into the portal veins without necessarily via secondary peritonitis. The most common comorbidities was peritonitis (52.4%) and hypertension (52.4%), followed by coronary heart disease (23.8%), diabetes (19.0%), and cerebrovascular disease (19.0%).

**Table 2:** Etiologies and comorbidities of 21 patients with HPVG.

Type	Value (%)
Etiologies	
Abdominal infection	2 (9.5)
Intestinal obstruction	3 (14.3)
Gastrointestinal bleeding	6 (28.6)
Gastrointestinal tumor	3 (14.3)
Gastrointestinal tumor with gastrointestinal bleeding	2 (9.5)
Gastrointestinal malignancy with liver metastasis	1 (4.8)
Postoperative gastrointestinal bleeding in pancreatic malignancy	1 (4.8)
Pancreatic malignancy with liver metastasis, obstructive jaundice	1 (4.8)
Intestinal necrosis	7 (33.3)
Mesenteric vascular embolism with intestinal necrosis	1 (4.8)
Intestinal bleeding with necrosis	4 (19.0)
Post-abdominal surgery	10 (47.6)
Postoperative gastrointestinal bleeding in abdominal malignancy	2 (9.5)
Post-abdominal surgery with abdominal infection	5 (23.8)
Post-abdominal surgery with intestinal obstruction	3 (14.3)
Post-abdominal surgery with abdominal infection and intestinal obstruction	2 (9.5)
Post-abdominal surgery with intestinal edema, adhesions	1 (4.8)
Peritonitis	11 (52.4)
Pathology report: intestinal necrosis (%)	4 (66.7)
Comorbidities	
Hypertension	11 (52.4)
Diabetes	4 (19.0)
Coronary heart disease	5 (23.8)
Cerebrovascular disease	4 (19.0)
Peritonitis	11 (52.4)
Mortality in patients with hypertension	3 (27.3)

### Predictor for surgical treatment and patient prognosis

Nine patients were recommended surgical treatment, but one patient refused surgery. The remaining 12 patients underwent conservative treatment. The survival rates of the surgical patients and patients who received conservative treatment were 75 and 76.9%, respectively. The overall in-hospital mortality rate was 23.8%. Among the five patients who died during hospitalization, four had intestinal necrosis, gastrointestinal bleeding, or both (Table 3). Patients were divided into two groups based on whether they underwent surgery, and various indicators were analyzed for each group (Table 4). We found significant differences between the surgical and conservative treatment groups in the following metrics: platelet (PLT) count (*p* = 0.005); HB in male patients (*p* = 0.009); neutrophils (*p* = 0.011); alanine transaminase (*p* = 0.027); aspartate aminotransferase (*p* = 0.014); and presence of hypertension (*p* = 0.024), peritonitis (*p* = 0.024), medication-relieved abdominal pain (*p* = 0.001), and colic (*p* = 0.024). We found that PLT (OR: 0.979; 95% CI: 0.962–0.997; *p* = 0.024) and neutrophil levels (OR: 1.161; 95% CI: 1.019–1.323; *p* = 0.025), and the presence of hypertension (OR: 15.750; 95% CI: 1.424–174.246; *p* = 0.025) and peritonitis (OR: 15.750; 95% CI: 1.424–174.246; *p* = 0.025) were significantly associated with the patients receiving surgical treatment (Table 5). It should be noted that these results are based on a small sample size of 21 patients, and thus, the associations should be interpreted with caution.

**Table 3:** Treatment and prognosis of patients with HPVG.

Variable	Value (%)
Number of patients eligible for surgery and without contraindications	9 (42.9)
Number of patients undergoing surgical treatment	8 (38.1)
Laparoscopic rectal cancer resection + left lateral sectionectomy of the liver + cholecystectomy	1 (4.8)
Subtotal resection of the entire small intestine and colon	1 (4.8)
Pylorus-preserving gastrectomy + gastrojejunostomy + duodenoplasty + bile duct repair	1 (4.8)
Right hemicolectomy + adhesiolysis	1 (4.8)
Distal ileal resection + partial ascending colon resection + ileostomy	1 (4.8)
Small intestine resection + right hemicolectomy + small bowel stoma	1 (4.8)
Laparotomy + hemostasis of intra-abdominal bleeding, followed by hepatic artery angiography	1 (4.8)
Number of patients eligible for surgery but not operated on	3 (14.3)
End-stage sigmoid colon adenocarcinoma, previous uremia, multi-organ failure	1 (4.8)
End-stage rectal cancer with gastrointestinal perforation and bleeding	1 (4.8)
Postoperative pancreatic malignancy, intestinal perforation and bleeding	1 (4.8)
Survival rate (%) of surgical patients	6 (75.0)
Number of patients receiving conservative treatment	13 (61.9)
Number of surviving patients and survival rate (%) of patients receiving conservative treatment	10 (76.9)
Number of patients cured and discharged	16 (76.2)
Number of patients who died in hospital or were discharged on their own accord	5 (23.8)
Number of patients who died after surgical treatment	2 (9.5)
Abdominal vascular embolization with extensive necrosis of gastrointestinal organs	1 (4.8)
Post-duodenal surgery duodenal fistula, severe infection	1 (4.8)
Number of patients who died after conservative treatment	3 (14.3)
Sigmoid colon adenocarcinoma with gastrointestinal bleeding, history of uremia	1 (4.8)
Rectal cancer with intestinal perforation, bleeding, and necrosis	1 (4.8)
Postoperative pancreatic malignancy, intestinal perforation and bleeding necrosis	1 (4.8)
Cause of death in deceased patients	
Malignant intestinal tumor with gastrointestinal bleeding	2 (9.5)
Gastrointestinal bleeding without intestinal necrosis	1 (4.8)
Intestinal necrosis without gastrointestinal bleeding	1 (4.8)
Intestinal bleeding and necrosis	2 (9.5)
With hypertension	4 (19.0)
Number of patients with non-end-stage malignant tumors	15 (71.4)
Number of surviving patients and survival rate of patients with non-end-stage malignant tumors	13 (86.7)
Number of patients with non-end-stage malignant tumors receiving surgical treatment	7 (46.7)
Number of surviving patients and survival rate of patients with non-end-stage malignant tumors receiving surgical treatment	5 (71.4)
Number of patients with non-end-stage malignant tumors receiving conservative treatment	8 (53.3)
Number of surviving patients and survival rate of patients with non-end-stage malignant tumors receiving conservative treatment	8 (100)

**Table 4:** Characteristics of patients undergoing surgery and conservative treatment.

Variable	Surgical treatment	Conservative treatment	*p* value
Age (years)	71.6 ± 15.1	55.5 ± 24.2	0.110
Sex (male/female)	4 (50.0%)/4 (50.0%)	9 (69.2%)/4 (30.8%)	0.646
RBC (Male/Female, 10^12^/L)	4.5 ± 0.6/3.2 ± 0.5	3.6 ± 1.3/3.4 ± 0.7	0.121/0.563
WBC (10^9^/L)	10.6 ± 5.1	9.0 ± 5.7	0.525
PLT (10^9^/L)	124.4 ± 81.0	219.6 ± 57.7	0.005
HB (Male/Female, g/L)	137.8 ± 17.5/96.3 ± 17.0	96.8 ± 22.6/100.0 ± 18.5	0.009/0.775
N (Neutrophil, %)	92.6 (80.8, 94.5)	71.3 (66.6, 77.9)	0.011
CRP (mg/L)	24.85 (5.8, 108.25)	70.0 (12.6, 109)	0.741
PCT (ng/L)	6.29 (0.37, 8.95)	0.14 (0.10, 0.43)	0.064
DD-I (D-Dimer, mg/L)	4,990 (3,370, 16,690)	4,540 (1,330, 9,175)	0.219
ALT (U/L)	62.5 (18.0, 3368.0)	21.0 (14.0, 57.0)	0.027
AST (U/L)	74.0 (33.0, 6965.8)	30.0 (19.0, 70.0)	0.014
Hypertension (yes/no)	7 (87.5%)/1 (12.5%)	4 (30.8%)/9 (69.2%)	0.024
Peritonitis (yes/no)	7 (87.5%)/1 (12.5%)	4 (30.8%)/9 (69.2%)	0.024
Intestinal necrosis (yes/no)	4 (50.0%)/4 (50.0%)	3 (23.1%)/10 (76.9%)	0.346
Intestinal obstruction (yes/no)	0 (0.0%)/8 (100.0%)	5 (38.5%)/8 (61.5%)	0.111
Medication-relievable abdominal pain (yes/no)	0 (0.0%)/8 (100.0%)	8 (80.0%)/2 (20.0%)	0.001
Colic (yes/no)	7 (87.5%)/1 (12.5%)	4 (30.8%)/9 (69.2%)	0.024
Gas accumulation are (within a liver segment/in two or more liver segments)	2 (25.0%)/6 (75.0%)	8 (61.5%)/5 (38.5%)	0.183

**Table 5:** Predictor for surgical treatment.

Variable	*β*	OR and 95%CI	*P* value
Age (years)	0.043	1.043 (0.988–1.102)	0.127
Sex (male/female)	−0.811	0.444 (0.072–2.740)	0.382
RBC (10^12^/L)	0.372	1.450 (0.622–3.383)	0.390
WBC (10^9^/L)	0.057	1.059 (0.896–1.251)	0.504
PLT (10^9^/L)	−0.021	0.979 (0.962–0.997)	0.024
HB (g/L)	0.038	1.038 (0.993–1.086)	0.101
N (Neutrophil, %)	0.149	1.161 (1.019–1.323)	0.025
CRP (mg/L)	−0.002	0.998 (0.983–1.014)	0.842
PCT (ng/L)	0.912	2.489 (0.413–15.005)	0.320
DD-I (D-Dimer, mg/L)	0.000	1.000 (1.000–1.000)	0.188
ALT (U/L)	0.025	1.025 (0.993–1.059)	0.129
AST (U/L)	0.019	1.019 (0.988–1.051)	0.233
Hypertension (yes/no)	2.757	15.750 (1.424–174.246)	0.025
Diabetes (yes/no)	1.974	7.200 (0.596–87.020)	0.121
CVD (yes/no)	0.105	1.111 (0.142–8.680)	0.920
Stroke (yes/no)	1.974	7.200 (0.596–87.020)	0.121
Peritonitis (yes/no)	2.757	15.750 (1.424–174.246)	0.025
Intestinal necrosis (yes/no)	1.204	3.333 (0.502–22.142)	0.213
Intestinal obstruction (yes/no)	−21.203	-	0.999
Gas accumulation are (within a liver segment/in two or more liver segments)	1.569	4.808 (0.682–33.333)	0.115

To eliminate the influence of end-stage tumors, we analyzed the data of patients without end-stage malignancies (Table S1). We found that there were statistically significant differences between the surgical and conservative treatment groups in the following metrics: PLT (*p* = 0.001); HB in male patients (*p* = 0.020); neutrophil (*p* = 0.001); aspartate aminotransferase (*p* = 0.049); presence of hypertension (*p* = 0.041), peritonitis (*p* = 0.007), intestinal necrosis (*p* = 0.026), medication-relieved abdominal pain (*p* = 0.001), and colic (*p* = 0.041); and the degree of gas accumulation (*p* = 0.041).

### Imaging analysis

Imaging findings of HPVG showed that branching gas accumulation can extend up to 2 cm within the liver capsule. Gas accumulation is typically caused by the retrograde flow of intestinal gas through the portal venous system into the liver. Patients with intestinal bleeding and necrosis often exhibit concurrent mesenteric venous gas accumulation, which indicates a close association between these complications and HPVG.

**Figure 1:**
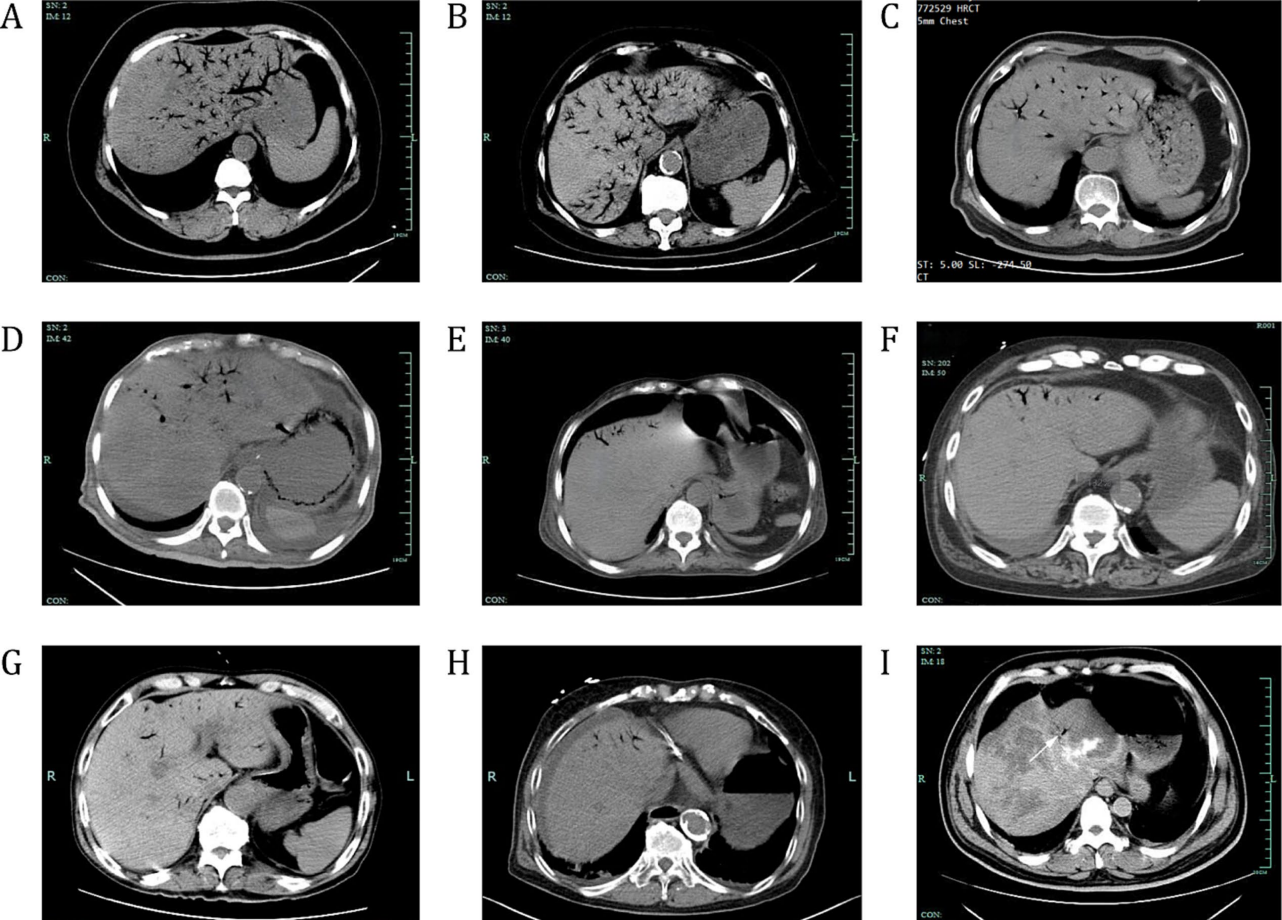
Nine patients with HPVG. CT scan of nine patients with varying degrees of HPVG. **(A,B)** Abdominal CT scans show extensive gas throughout the entire portal venous system. ‘Entire’ indicates that gas is present in two or more hepatic segments, with the gas occupying a relatively large area within these segments. **(C–G)** Gas is present in more than two segments of the liver. ‘Two segments’ means that gas is present in two or more hepatic segments, but the area occupied by the gas within each of these segments is relatively small. **(H,I)** Minimal gas is found within a 2 cm range of the liver capsule or within a single liver segment. HPVG, hepatic portal venous gas; CT, computed tomography.

The radiological manifestations are detailed in Figure 1 to better understand and identify the various characteristics of HPVG. Nine different radiological manifestations of HPVG were identified: (1) extensive gas presence throughout the portal venous system, (2) gas present in more than two hepatic segments, and (3) minimal gas present within 2 cm of the liver capsule or in a single hepatic segment.

**Figure 2:**
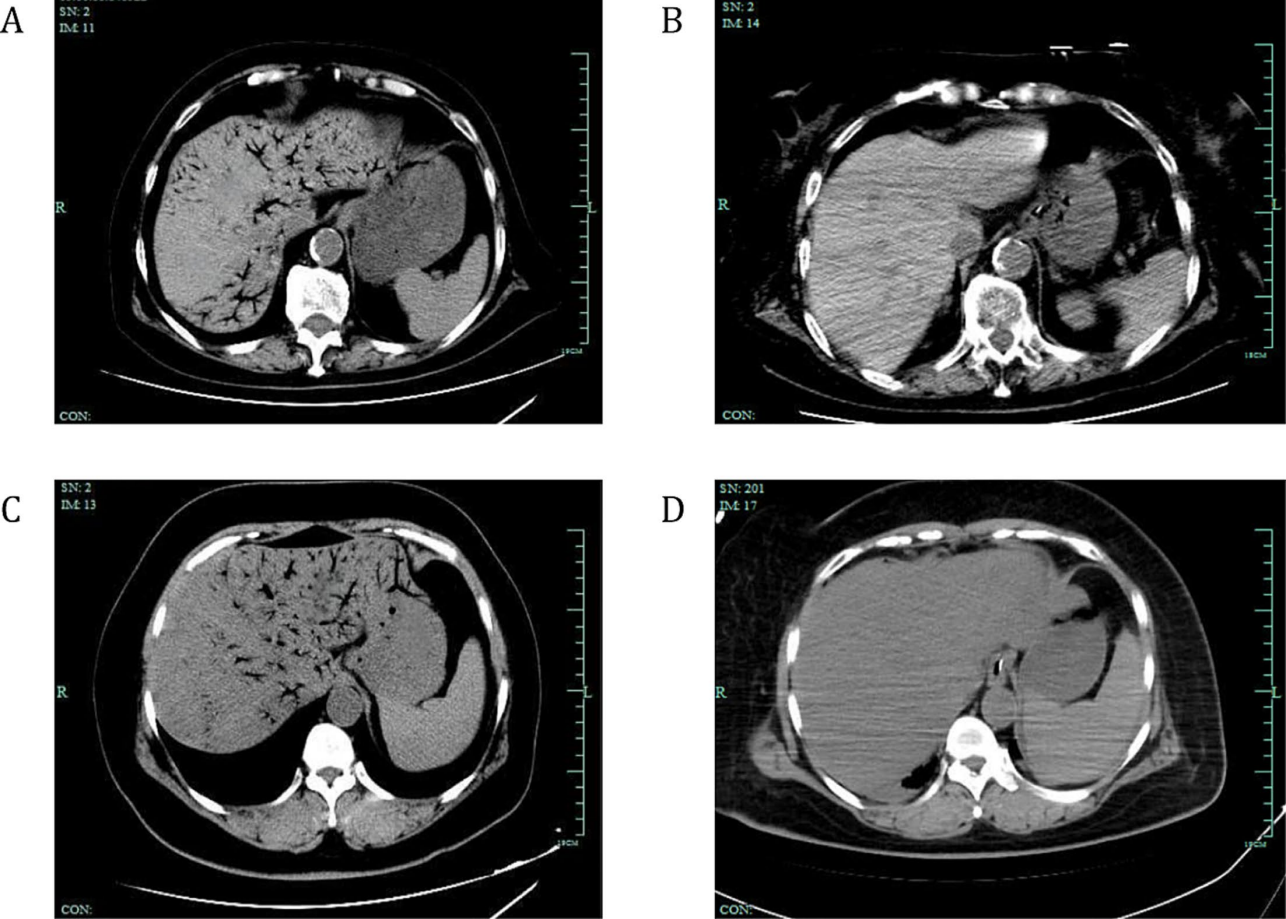
Changes in gas absorption before and after surgical treatment in two patients with HPVG. CT scans showing the absorption of venous gas in two surgically-treated patients with HPVG. **(A)** The abdominal CT scan displays extensive gas within the portal vein system and mesenteric veins. **(B)** Two days later, the gas dissipated. **(C)** Another abdominal CT scan showing extensive gas within the portal vein system and mesenteric veins. **(D)** One day later, the gas dissipated. White arrows indicate the presence of gas within the venous system. HPVG, hepatic portal venous gas; CT, computed tomography.

To further illustrate the treatment outcomes of patients with HPVG, Figure 2 presents the CT scans of two patients with varying degrees of HPVG before and after surgical treatment. These images show the pre- and post-surgical comparisons, as well as the changes in gas absorption. These images showed that surgical treatment significantly improved gas accumulation and recovery after surgery.

## Discussion

This study showed that various gastrointestinal diseases and invasive treatments can lead to HPVG. The prognosis of these patients is generally favorable, and even in those with bowel obstruction, the mortality rate can be significantly reduced if effective treatment measures are taken before extensive bowel necrosis or severe signs of infection (17). The diagnostic and treatment modalities for HPVG have undergone various changes, and this study systematically analyzed the clinical characteristics, etiologies, surgical indications, and outcomes of patients with HPVG. We found that the main causes of HPVG included peritonitis, post-abdominal surgery intestinal necrosis, and gastrointestinal bleeding. The common comorbidities in these patients were primarily peritonitis and hypertension. The results showed no statistically significant difference in survival rates between patients who underwent surgical treatment and those who received conservative treatment; however, the decision to perform surgery was primarily influenced by the PLT count, neutrophil level, and presence of hypertension and peritonitis.

We found that patients with HPVG had reduced levels of RBC and HB, and significantly elevated levels of WBC, CRP, and DD-I. These results indicate that the occurrence of HPVG is closely related to the induction of inflammation and vascular damage, which may serve as trigger points for HPVG. Intestinal ischemia and necrosis lead to the death of intestinal wall cells and release of gas (18); bacterial infections and gas-producing bacteria in the intestine produce large amounts of gas (19); mechanical injuries, such as abdominal surgery, cause damage to the intestinal wall or blood vessels that induce inflammatory responses, which increase vascular permeability, allowing gas to more easily pass through the vessel walls into the portal venous system (20); and conditions such as portal hypertension and gastrointestinal perforation allow gas to directly enter the circulation (21, 22). Therefore, elevated CRP, WBC, and DD-I levels along with reduced RBC and HB levels may indicate the presence of HPVG.

The main causes of HPVG include peritonitis, intestinal necrosis after abdominal surgery, and gastrointestinal bleeding. The primary reasons for these conditions are as follows: (1) peritonitis-induced inflammatory responses can increase vascular permeability, allowing gas to pass more easily through damaged vessel walls into the circulation (23); (2) abdominal surgery can cause mechanical damage to the intestinal wall or blood vessels, providing a pathway for gas to enter the portal venous system (24); (3) intestinal necrosis leads to the death of intestinal wall cells and the release of gas (25), while gastrointestinal bleeding may be accompanied by inflammation and tissue damage, further promoting gas production and diffusion (26). The main comorbidities of patients with HPVG include hypertension, coronary heart disease, diabetes, and cerebrovascular disease, which are often associated with chronic inflammatory states and vascular damage that increase the risk of HPVG. Hypertension and coronary heart disease can lead to increased fragility and permeability of the vascular walls, making it easier for gases to pass through the damaged vessels into the portal venous system (27). In patients with diabetes, high blood glucose levels can cause microvascular changes and inflammatory responses, further exacerbating this process (28). Cerebrovascular disease may also be associated with systemic inflammatory responses, thereby increasing the risk of HPVG (29). The presence of these comorbidities not only affects the overall health of patients but also plays a significant role in the occurrence and progression of HPVG.

Recently, the mortality rate of patients with HPVG has decreased to less than 50% (30, 31), but patients with ischemic bowel necrosis still exhibit a high mortality rate, typically exceeding 50% (9). In this study, of the eight patients who underwent surgical treatment, six (75%) survived and were discharged. Although Kinoshita et al. reported that the involvement of three or more branches of the portal vein is a significant marker of potentially fatal outcomes and is associated with a poor prognosis in 75% of cases (12), our study found different results, wherein the mortality rate for patients with extensive portal venous gas showing the typical tree-branching pattern was only 20%. After postoperative treatment, the cure rate for these patients was 100%, with only one patient dying because of refusal to undergo surgery. Moreover, although our initial analysis did not strictly adhere to the three-or-more-regions versus fewer-affected-regions division, we found that patients with gas accumulation in two or more segments, which may be related to the concept of three or more regions, had a higher prevalence of severe underlying conditions. Of the 13 patients who received conservative treatment, 10 survived and were discharged. Among the fatal cases, 80% had varying degrees of ischemic bowel necrosis. In the postoperative pathological reports of the six cases, four (66.7%) documented bowel necrosis, with two cases resulting in in-hospital deaths. Even in the absence of signs of bowel necrosis, conditions such as bowel obstruction and gastrointestinal tumors should receive high clinical attention because these conditions can pose a risk for ischemic necrosis. Among the 11 patients with HPVG and symptoms of peritonitis, the mortality rate for the four patients who received conservative treatment was 50%, while the mortality rate for the seven patients who underwent surgical treatment was 28.6%. Although there was no statistically significant difference in survival rates between the surgical and conservative treatment groups in this study, the degree of venous gas accumulation was more severe in surgically treated patients than in those who received conservative treatment.

We found that the decision to choose surgical treatment was influenced by the PLT count, neutrophil levels, and the presence of hypertension and peritonitis. Thrombocytopenia may indicate severe inflammation, infection, or coagulation disorders, which are often signs of serious underlying conditions (32). Elevated neutrophil levels typically suggest inflammation or infection, and surgical intervention can help reduce the systemic inflammatory response and prevent organ dysfunction (23). Hypertension is not only a potential risk factor for HPVG but also a risk factor for poor patient prognosis; therefore, patients with HPVG and hypertension may require aggressive surgical intervention. Patients with signs of peritonitis are at a higher risk of intestinal necrosis and mortality; thus, the presence of peritoneal irritation and significantly elevated serum inflammatory markers may indicate the need for surgical treatment. For patients with stable postoperative HPVG, attention should be paid to the absorption of portal venous gas and a follow-up CT scan should be performed if necessary.

Our findings suggest that while comorbidities are important considerations, they should not be the only factors dictating the treatment approach. The survival of patients with comorbidities who underwent surgery indicates that a more comprehensive assessment, including the severity of the underlying condition causing HPVG, the patient’s overall physiological reserve, and the potential benefits of surgery in alleviating the acute problem, is necessary. For example, in cases where the underlying cause of HPVG, such as intestinal necrosis, is likely to progress rapidly and lead to a worse outcome without surgical intervention, the presence of comorbidities may not be an absolute contraindication. Instead, appropriate pre-operative optimization and peri-operative management can be implemented to improve the chances of a successful surgical outcome.

In our analysis, we explored the relationship between gas accumulation patterns and intestinal necrosis. Patients with intestinal necrosis frequently exhibited specific gas accumulation patterns. Intestinal necrosis results in the death of intestinal wall cells, leading to gas release that enters the portal venous system. A common finding was the co-occurrence of mesenteric venous gas accumulation along with gas in the hepatic portal venous system. This is likely a consequence of the compromised intestinal wall integrity, allowing gas to infiltrate adjacent mesenteric vessels that drain into the portal vein. Moreover, extensive intestinal necrosis was often associated with a more widespread and prominent gas distribution in the portal venous system. The branching pattern of gas was more pronounced, often extending to multiple hepatic segments. This could be attributed to the larger amount of gas produced by a greater area of necrotic intestine, which is then transported to the portal venous system. Regarding the relationship between gas distribution and prognosis, our data indicated that a wider distribution of gas in the hepatic portal venous system was associated with a higher likelihood of intestinal necrosis, which is a key determinant of poor prognosis. In our study, among patients with extensive gas distribution, a significant proportion had intestinal necrosis. In fact, 80% of the fatal cases had varying degrees of ischemic bowel necrosis, and these cases commonly presented with more widespread gas accumulation.

Our study is significantly limited by the small sample size of only 21 patients. This has directly affected the statistical power of our analysis, making it challenging to precisely evaluate the relationships between blood parameters, comorbidities, and the need for surgical intervention. For example, the associations we found between platelet count, neutrophil levels, hypertension, and peritonitis and the likelihood of surgical treatment may be spurious due to the small number of observations. As a result, the conclusions drawn from this study are tentative and should not be considered definitive. Future research with larger, multi-center studies is essential to confirm or refute our findings and to provide more robust evidence-based guidelines for the management of HPVG.

## Conclusion

In conclusion, this retrospective analysis of 21 patients with HPVG revealed several important findings. The primary causes of HPVG were identified as peritonitis (52.4%), post-abdominal surgery (47.6%), intestinal necrosis (33.3%), and gastrointestinal bleeding (28.6%). The common comorbidities among these patients included peritonitis (52.4%), hypertension (52.4%), and coronary heart disease (23.8%). Platelet count, neutrophil levels, presence of hypertension, and peritonitis showed associations with the likelihood of surgical intervention, yet these findings require validation in larger cohorts.

Based on our study results, there is a clear need to reassess patient selection criteria for surgical intervention in patients with HPVG. Future studies should focus on developing a more comprehensive and evidence-based algorithm that takes into account multiple factors, including comorbidities, the underlying etiology of HPVG, the severity of symptoms such as abdominal pain and its response to treatment, and the patient’s overall physiological status. This will help in making more accurate decisions regarding surgical vs. conservative management, ultimately improving patient outcomes.

## Data Availability

The original contributions presented in the study are included in the article/Supplementary material, further inquiries can be directed to the corresponding author/s.
