# Supplementary material for: Clinical characteristics and treatment of hepatic portal venous gas: case series and literature review
Source: Front Med (Lausanne). 2025 May 9;12:1540418. doi: 10.3389/fmed.2025.1540418 (PMC12098563; doi:10.3389/fmed.2025.1540418)
Supplement: Supplementary file 1 [file Table_1.docx]

Table S1. Indicators of patients with non-advanced tumors undergoing surgery and conservative treatment

| Variable | Surgical treatment | Conservative treatment | *P* value |
| --- | --- | --- | --- |
| Age (years) | 70.0±15.5 | 48.6±27.3 | 0.091 |
| Sex (male/female) | 3 (42.9%)/4 (57.1%) | 5 (62.5%)/3 (37.5%) | 0.619 |
| RBC (Male/Female, 10^12^/L) | 5.0±0.4/3.2±0.5 | 4.4±1.2/3.8±0.2 | 0.472/0.114 |
| WBC (10^9^/L) | 10.2±5.3 | 6.8±4.7 | 0.199 |
| PLT (10^9^/L) | 100.0±45.9 | 233.5±54.2 | 0.001 |
| HB (Male/Female, g/L) | 143.3±16.5/96.3±17.0 | 114.8±9.9/107.0±14.7 | 0.020/0.423 |
| N (Neutrophil, %) | 92.4 (80.5, 93.3) | 71.2 (64.1, 77.2) | 0.001 |
| CRP (mg/L) | 23.7 (4.4, 116.0) | 16.3 (1.0, 75.5) | 0.475 |
| PCT (ng/L) | 5.46 (0.30, 9.39) | 0.12 (0.08, 0.22) | 0.086 |
| DD-I (D-Dimer, mg/L) | 3720 (3360, 24822) | 4420 (610, 5440) | 0.156 |
| ALT (U/L) | 22.0 (17.0, 6305.0) | 19.0 (11.0, 51.0) | 0.056 |
| AST (U/L) | 37.0 (29.0, 13276.5) | 30.0 (18.0, 55.0) | 0.049 |
| Hypertension (yes/no) | 6 (85.7%)/1 (14.3%) | 2 (25.0%)/6 (75.0%) | 0.041 |
| Peritonitis (yes/no) | 7 (100.0%)/0 (0.0%) | 2 (25.0%)/6 (75.0%) | 0.007 |
| Intestinal necrosis (yes/no) | 4 (57.1%)/3 (42.9%) | 0 (0.0%)/8 (100.0%) | 0.026 |
| Intestinal obstruction (yes/no) | 0 (0.0%)/7 (100.0%) | 2 (25.0%)/6 (75.0%) | 0.467 |
| Medication-relievable abdominal pain (yes/no) | 0 (0.0%)/7 (100.0%) | 7 (87.5%)/1 (12.5%) | 0.001 |
| Colic (yes/no) | 6 (85.7%)/1 (14.3%) | 2 (25.0%)/6 (75.0%) | 0.041 |
| Gas accumulation are (within a liver segment/in two or more liver segments) | 2 (28.6%)/5 (71.4%) | 7 (87.5%)/1 (12.5%) | 0.041 |
